# Age-related increases in false recognition: the role of perceptual and conceptual similarity

**DOI:** 10.3389/fnagi.2014.00283

**Published:** 2014-10-17

**Authors:** Laura M. Pidgeon, Alexa M. Morcom

**Affiliations:** Department of Psychology, Centre for Cognitive Ageing and Cognitive Epidemiology, University of EdinburghEdinburgh, UK

**Keywords:** cognitive aging, episodic memory, false recognition, pattern separation, gist, perceptual similarity, conceptual similarity

## Abstract

Older adults (OAs) are more likely to falsely recognize novel events than young adults, and recent behavioral and neuroimaging evidence points to a reduced ability to distinguish overlapping information due to decline in hippocampal pattern separation. However, other data suggest a critical role for semantic similarity. Koutstaal et al. [(2003) false recognition of abstract vs. common objects in older and younger adults: testing the semantic categorization account, *J. Exp. Psychol. Learn.* 29, 499–510] reported that OAs were only vulnerable to false recognition of items with pre-existing semantic representations. We replicated Koutstaal et al.’s (2003) second experiment and examined the influence of independently rated perceptual and conceptual similarity between stimuli and lures. At study, young and OAs judged the pleasantness of pictures of abstract (unfamiliar) and concrete (familiar) items, followed by a surprise recognition test including studied items, similar lures, and novel unrelated items. Experiment 1 used dichotomous “old/new” responses at test, while in Experiment 2 participants were also asked to judge lures as “similar,” to increase explicit demands on pattern separation. In both experiments, OAs showed a greater increase in false recognition for concrete than abstract items relative to the young, replicating Koutstaal et al.’s (2003) findings. However, unlike in the earlier study, there was also an age-related increase in false recognition of abstract lures when multiple similar images had been studied. In line with pattern separation accounts of false recognition, OAs were more likely to misclassify concrete lures with high and moderate, but not low degrees of rated similarity to studied items. Results are consistent with the view that OAs are particularly susceptible to semantic interference in recognition memory, and with the possibility that this reflects age-related decline in pattern separation.

## INTRODUCTION

Older adults (OAs) are more prone to false recognition than young adults (YAs), particularly when novel events are similar to those previously encountered (Koutstaal and Schacter, 1997; Yassa et al., 2011a). This suggests that a fundamental feature of recognition impairments in older individuals is reduced ability to discriminate in memory (mnemonic discrimination) between similar representations (Stark et al., 2013; Yeung et al., 2013). Electrophysiological and neuroimaging data support proposals that age-related impairments of mnemonic discrimination reflect reduced capacity to pattern separate (orthogonalise) incoming representations, leading to greater overlap between memory representations (Wilson et al., 2006). Pattern separation accounts predict that many kinds of representational overlap are less efficiently discriminated in older age, without specifying a unique role for semantic overlap. However, it has also been suggested that increased false recognition with age is due to greater emphasis on processing of *gist*, particularly semantic gist (Koutstaal and Schacter, 1997; Tun et al., 1998). Koutstaal et al.’s (2003) *semantic categorization account* makes the specific proposal that increases in false recognition are due to greater emphasis by OAs on semantic processing at encoding. To evaluate these accounts it is critical to establish whether the age-related increase in false recognition is in fact driven specifically by semantic similarity.

False recognition of words and images increases markedly with age. In the Deese–Roediger–McDermott paradigm (Deese, 1959; Roediger and McDermott, 1995), OAs falsely recognize up to 80% of critical lure words which are strongly associated with lists of studied words, compared to up to 65% among YAs (Norman and Schacter, 1997). In paradigms employing visual images, OAs falsely recognize perceptually similar images from the same basic-level conceptual category as studied images (e.g., *cats*) up to 35% more often than YAs (Koutstaal and Schacter, 1997; Koutstaal et al., 1999a). Such findings have been described in terms of greater reliance among OAs on semantic gist (general representations of meaning; Brainerd and Reyna, 2002), leading to heightened false recognition where lure items overlap with studied items in meaning or conceptual category (Norman and Schacter, 1997; Reyna and Brainerd, 1998; Tun et al., 1998). In support of this, Dennis et al. (2007, 2014) report functional magnetic resonance imaging (fMRI) evidence of increased semantic processing among OAs at encoding and during recognition. Moreover, studying multiple category exemplars is thought to strengthen semantic gist representations: larger effects of category size on false recognition have been reported in OAs, supporting the notion of increased reliance on semantic gist representations with age (Koutstaal and Schacter, 1997).

Although most gist-based accounts emphasize the role of overlapping semantic information in driving false recognition, they do not discount the notion that other kinds of similarity can contribute to recognition outcomes. Perceptual gist representations (general perceptual representations based on overall shape, color etc.) are also thought to be strengthened with exposure to multiple visually similar items (Koutstaal et al., 1999b), but effects of perceptual similarity on false recognition have been considered to be equivalent in young and OAs (Schacter et al., 1997; Koutstaal et al., 1999b; see also Budson et al., 2003). To our knowledge only one study has compared perceptual and semantic similarity effects on false recognition in healthy aging (Koutstaal et al., 2003). Noting that in recognition studies employing visual images, lures typically show both conceptual (category membership) and perceptual (shape, color) overlap with studied items, Koutstaal et al. (2003) employed unfamiliar abstract shapes grouped into categories based on visual similarity to examine whether age-related increases in false recognition result only from semantic gist, or can be driven by perceptual similarity. When verbal conceptual labels were assigned to abstract categories at both study and test, OAs were more likely to show false recognition than when no labels were provided. In a second experiment, OAs were more likely than YAs to falsely recognize concrete, meaningful images sharing basic-level category membership with studied images, but were not more likely to falsely recognize abstract lures (sharing only perceptual features with studied items). This effect of conceptual information was present even when many perceptually similar abstract category members were presented, suggesting age-related increases in false recognition are influenced by semantic but not perceptual gist, consistent with the semantic categorization account (Koutstaal et al., 2003).

Pattern separation accounts do not place special emphasis on semantic information, instead pointing to age-related impairments in mnemonic discrimination along multiple dimensions of similarity. This is proposed to be due to decline in hippocampal pattern separation; the formation of unique neural representations from incoming sensory input, minimizing overlap with existing representations (Wilson et al., 2003, 2006). Rodent electrophysiological and human fMRI data suggest advancing age is associated with rigidity of hippocampal neuronal responses, with a shift from a tendency to pattern separate incoming representations toward a tendency for pattern completion, i.e., reinstatement of existing representations based on incomplete cues. It has been suggested that this decline in pattern separation contributes to age-related reductions in the capacity for mnemonic discrimination (Wilson et al., 2003; Yassa et al., 2011a,b). Although by definition an encoding process, pattern separation can be elicited at retrieval if there is interference between incoming information and to-be-retrieved representations, and therefore can contribute to mnemonic discrimination outcomes at encoding and/or retrieval (Yassa and Stark, 2011). Behavioral investigations suggest that OAs are less likely to correctly reject as “similar” lures which are perceptually and conceptually similar to studied items, and more likely to falsely recognize lures as “old” (Toner et al., 2009; Stark et al., 2013). The degree of this impairment in mnemonic discrimination has been found to correlate with age-related structural and functional changes in the hippocampus, supporting assumptions that less efficient pattern separation contributes to decline in mnemonic discrimination (Yassa et al., 2011a,b). Age-related impairments have also been demonstrated in mnemonic discrimination of lure and studied items presented in close temporal or spatial proximity over short retention intervals of less than 1 min (Stark et al., 2010; Holden et al., 2012; Tolentino et al., 2012), and Reagh et al. (2014) showed spatial discrimination deficits over delays up to 12 min, similar to intervals typically employed in recognition studies. Spatial discrimination is also impaired in aged rodents (e.g., Wilson et al., 2003). A study employing visually presented verbal stimuli reported age-related impairments in mnemonic discrimination of perceptually similar, but not conceptually similar words (Ly et al., 2013).

Age-related decline in pattern separation with increased bias toward pattern completion has been proposed as a potential mechanism for the age-related increase in gist-based false recognition as well as for reductions in mnemonic discrimination along multiple dimensions of overlap (Schacter et al., 1998; Yassa et al., 2011a). However, any integration of pattern separation and gist accounts requires specification of the role of semantic information, which is central to gist accounts (Norman and Schacter, 1997; Reyna and Brainerd, 1998; Tun et al., 1998). Moreover, the semantic categorization account (Koutstaal et al., 2003) proposes a specific mechanism for greater gist reliance in aging. If OAs explicitly process conceptual information at encoding at the expense of perceptual detail, false recognition increases will be driven specifically by semantic relatedness, and perceptual similarity will have, if anything, a reduced effect. Conversely, a decline in neural pattern separation predicts a more general impact on mnemonic discrimination and gist reliance in OAs which extends beyond semantic gist. Human behavioral investigations of mnemonic discrimination have suggested such a general decline, unlike Koutstaal et al.’s (2003) findings, but it is possible that use of images of meaningful objects (Stark et al., 2010; Yassa et al., 2011a), or nameable shapes (Tolentino et al., 2012) has meant that estimates of age-related impairments in spatial, temporal, or perceptual discrimination are influenced by semantic content.

The present study investigated whether age-related increases in false recognition are dependent on overlapping conceptual representations, as well as evaluating the relative contributions of perceptual and conceptual similarity. In two experiments, we sought to replicate and extend findings of Koutstaal et al. (2003), using the original study’s abstract and concrete images. Experiment 1 employed an “old/new” recognition paradigm in a direct replication of Koutstaal et al.’s (2003) Experiment 2, with one modification. In the earlier study, multiple exemplars from each studied category were presented at test and at study, making it difficult to separate study phase and test phase interference, and possibly leading to elevated false recognition in both age groups by increasing bias toward responding “old,” lessening age differences. Thus, during the test phase we included a single studied item and single lure for each category which was encountered in the study phase. Age-related increases in false recognition of concrete but not abstract lures would provide support for a uniquely semantic gist-based account of false recognition in aging, such as the semantic categorization account (Koutstaal et al., 2003). Increased false recognition of abstract as well as concrete lures would however indicate impaired mnemonic discrimination along multiple domains of similarity, as proposed by pattern separation models and also consistent with a more generalized gist-based account. If perceptual gist as well as semantic gist influences false recognition in old age, effects of study phase category size would be expected to be larger among OAs for both stimulus types.

Experiment 2 was identical to Experiment 1, but participants were asked to respond “old,” “similar” or “new” to studied, lure and novel items respectively, instead of simply “old/new.” The additional “similar” response option has been found to reduce gist-based false recognition in young and OAs (Koutstaal et al., 1999a), and is thought to place greater demands on pattern separation (Stark et al., 2013). Based on previous findings, it was predicted that OAs would show greater false recognition and reduced correct rejection of lures (Stark et al., 2013). As in Experiment 1, if older age is associated with general mnemonic discrimination decline, this pattern was expected for abstract and concrete items, whereas the semantic categorization account predicted age differences only for concrete items.

We also sought to investigate the prediction from pattern separation models that OAs require greater change in input in order to successfully discriminate lures from studied items (Wilson et al., 2006), using measures of subjectively rated within-category perceptual and conceptual similarity (Konkle et al., 2010). These measures provided a further test of the role of conceptual similarity in age-related increases in false recognition. If this is critical in driving age-related increases in false recognition, as assumed by the semantic categorization account, OAs would be expected to show greater effects of conceptual similarity on false recognition, while perceptual similarity effects would be equivalent in the two age groups.

## MATERIALS AND METHODS

### PARTICIPANTS

Demographic and cognitive test data for participants in Experiments 1 and 2 are shown in **Table 1**. We estimated that 24 participants per group were required to replicate Koutstaal et al.’s (2003) critical Stimulus Type × Age interaction with 95% power (G^∗^Power; Erdfelder et al., 1996), following correction for bias true effect size estimation (Tversky and Kahneman, 1971; ). Twenty-four YAs (aged 18–26 years) and 24 OAs (aged 60–79) took part in Experiment 1. A further YA was excluded from analyses due to incomplete data. Twenty-six YAs (aged 18–28) and 26 OAs (aged 62–79) participated in Experiment 2. Data from one additional OA were excluded due to failure to use the “similar” response during the test phase. All participants completed the following baseline cognitive tests: the Wechsler Test of Adult Reading (WTAR; Wechsler, 2001), and the Digit Symbol Coding and Digit Span Forward and Backward subscales of the Wechsler Adult Intelligence Scale IV (WAIS-IV^UK^; Wechsler, 2008). Raw WTAR scores were converted to Standard Scores based on the UK Standardization Sample.

**Table 1:** Demographic and cognitive test data for participants from Experiments 1 and 2.

	Experiment 1		Experiment 2	
	YA (*n* = 24)	OA (*n* = 24)	YA (*n* = 26)	OA (*n* = 26)
Age	20.6 (2.0) ^a^	70.5 (5.6) ^a^	21.6 (2.8) ^a^	69.2 (4.4) ^a^
Sex (*N* female)	13	12	21 ^a^	14 ^a^
Years of education	15.6 (1.6)	16.6 (3.5)	16.0 (2.0)	15.9 (4.2)
WTAR(Standard Score)	122 (5.7) (*n* = 19)	120 (7.1)	117 (6.8) (*n* = 16)	118 (6.3)
Digit Symbol	63.5 (10.4) ^a^	46.5 (10.8) ^a^	67.5 (8.7) ^a^	50.7 (13.4) ^a^
Digit Span Forward	7.6 (1.0)	7.3 (1.0)	7.2 (1.1)	7.5 (1.1)
Digit Span Backward	5.8 (1.1)^b^	5.6 (1.1)	4.9 (1.2) ^b^	5.3 (1.2)

*Mean (standard deviation). ^a^ Denotes significant within-experiment age difference (*p* < 0.05). ^b^Denotes significant difference between Experiments 1 and 2. WTAR, Wechsler Test of Adult Reading. WTAR scores for non-native English speakers were excluded. See section “Cognitive Tests” for details of statistical analyses.*

A separate sample of 24 OAs (aged 60–75) and 24 YAs (aged 18–25) provided subjective ratings of stimuli employed in Experiments 1 and 2. Half gave perceptual similarity ratings and half gave conceptual similarity ratings for all categories. Two YAs and one OA were excluded from analyses of conceptual and perceptual ratings respectively, as the average correlation of their ratings with the remainder of the sample was >2 SDs from the mean sample correlation (Konkle et al., 2010). Conceptual ratings were therefore based on 10 YAs and 12 OAs, and perceptual ratings on 12 YAs and 11 OAs.

All experimental procedures were approved by the Psychology Research Ethics Committee of the University of Edinburgh. Informed consent was obtained, and all participants were fully debriefed following completion of the experiment.

### STIMULI

**FIGURE 1:**
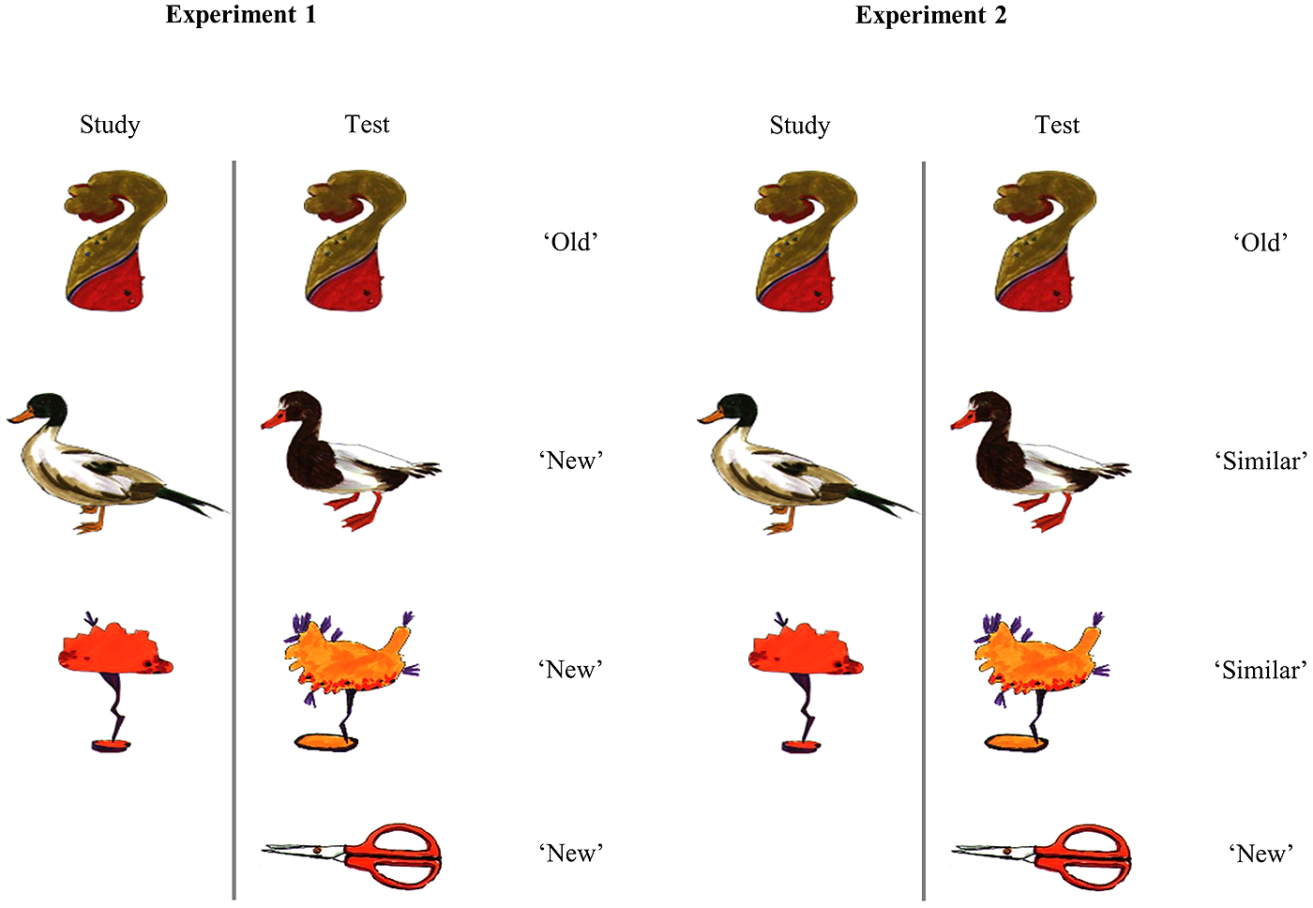
**Examples of study and test stimuli employed in Experiment 1 (test response options “Old” and “New”) and Experiment 2 (test response options “Old,” “Similar” and “New,” and the correct response associated with each image at test.** Stimuli taken from Koutstaal et al. (2003).

Stimuli were colored line drawings of categorized abstract and concrete items, employed by Koutstaal et al. (2003; see **Figure 1** for examples). Abstract items were unfamiliar shapes grouped into categories based on perceptual features, e.g., shape, color. Concrete items were drawings of familiar objects and animals grouped into categories according to basic-level conceptual category, e.g., *hats, ducks*. Categories consisted of 2 or 13 exemplars. Thirteen-exemplar categories were employed during the study phase as either single or large categories, and exemplars were presented as studied items and lures during the test phase. Two-exemplar categories were employed as novel categories during the test phase.

In both experiments, concrete items presented at study included 12 large categories (nine items presented per category) and 12 single-item categories (one exemplar). Thus, a total of 108 large category concrete items (nine exemplars from 12 categories) and 12 single category concrete items (one exemplar from 12 categories), were presented at study. The same distribution applied to abstract items (108 large category items; 12 single category items). Test phase lists comprised 48 studied items (of which 24 had been presented as part of large categories at study; 24 as single exemplars); 48 similar lures, i.e., novel images from studied categories (24 from large categories; 24 from single categories), and 48 novel items from 48 novel categories. Half of the items in each of these stimulus conditions were abstract, half were concrete. The stimulus condition of exemplars (studied or lure) was counterbalanced across participants. During study and test phases, abstract and concrete stimuli were intermixed and a unique pseudorandom order of presentation was generated for each participant.

### PROCEDURE

#### Experiment 1

Experiment 1 consisted of a study phase, followed by a 10 min filled interval, before a surprise recognition test. Study and test procedure followed Koutstaal et al.’s (2003) Experiment 2. During the study phase, participants viewed 240 images, and were asked to rate how pleasant they found each image from 1 to 5 (1 = very unpleasant; 5 = very pleasant). Images were ∼350 × 350 pixels and were viewed at a distance of around 50 cm on a PC screen, against a white background. Images were presented for 3 s, with a black fixation cross presented for 1.5 s between trials.

The test phase followed a 10 min interval during which cognitive tests were completed (see Participants). During the test phase, participants viewed 144 images, and judged each as “old” or “new” using key presses (**Figure 1**). Images were presented for 3.5 s, followed by a fixation cross for 1 s. Following each trial, participants were prompted to rate their confidence in their response, from 1 (just guessing) to 5 (very confident) using number keys. The prompt was presented for 4 s followed by a fixation cross for 0.8 s before the start of the next trial.

#### Experiment 2

Experiment 2 differed from Experiment 1 only in the test phase task instructions and response options; stimuli and study phase procedure were as in Experiment 1. Participants were informed that during the recognition test, items would either be identical to studied items, novel but similar to studied items, or novel and unrelated to studied items. Participants were asked to respond “old,” “similar,” or “new” accordingly (**Figure 1**). As in Experiment 1, participants then rated their confidence in their responses. Study phase timings were as in Experiment 2. During the test phase, stimulus presentation timings were as in Experiment 1 for all YAs. However, as the initial three OAs struggled to give recognition responses within 3.5 s, on average responding to only 65% of trials, presentation time was increased to 4.5 s for the remaining OAs. For the first 3 OAs, responses given within 4.5 s were retrieved from log files and recorded: their pattern of performance did not differ from the other OAs. For both age groups, the confidence rating prompt was presented for up to 4 s, or until 0.8 s after a response was made, followed by a fixation cross for 0.8 s before the next trial.

#### Ratings task

Participants in the ratings task gave either perceptual or conceptual similarity ratings for all categories (abstract and concrete). Images were reduced to ∼150 × 150 pixels, and all exemplars of a given category were presented simultaneously, against a white background.

During the perceptual task, participants rated the overall visual similarity of items in each category from 1 (very similar) to 5 (very distinctive) using the number keys. Participants were asked to base judgments on visual features only, e.g., shape, color. Abstract and concrete categories were presented together in a single block, with the order of category presentation randomized across participants. Images remained on screen until a response was made.

In the conceptual task, concrete and abstract images were presented in separate blocks, the order of which was counterbalanced across participants. For concrete images, participants were asked to rate categories according to how many *kinds* of object were present (1 = few kinds; 5 = many kinds), following Konkle et al. (2010). For example, a category of ducks comprising several distinct breeds of duck would be considered to contain more kinds than a category of apples containing only red apples. This provided a measure of within-category conceptual similarity. As abstract items by definition were not necessarily conceptually meaningful, for these items a modified conceptual ratings task was used. Participants were presented with abstract categories in sequence, and were asked firstly to provide a verbal label of a concrete object which they perceived some or all category members to resemble, and secondly to rate from 1 to 5 the ease of assigning a label that fit all category exemplars. This measure was assumed to reflect the fit of conceptual labels within each abstract category, equivalent to conceptual similarity. Ratings were inverted so that low scores reflected greater similarity, in line with the perceptual scale.

The 13-exemplar categories used as studied or lure items at test were divided into tertiles representing high, medium, and low conceptual and perceptual similarity on the basis of average ratings, separately for abstract and concrete items. For both experiments, mean proportion of lures falsely recognized by each participant at each level of similarity was calculated, separately for abstract and concrete stimuli and for perceptual and conceptual ratings. In each experiment, proportions for each participant were calculated across nine high, eight medium, and seven low perceptual similarity abstract lures, and across seven high, nine medium, and eight low perceptual similarity concrete lures. For conceptual similarity, mean proportions were calculated from 8 abstract and eight concrete lures at each level of similarity.

## RESULTS

In all analyses of variance (ANOVAs), Greenhouse–Geisser corrected degrees of freedom and *p* values are reported in cases where Mauchly’s test for violation of the assumption of sphericity was significant. In analyses of recognition performance, “highly confident” refers to responses receiving a confidence rating of 4 or 5.

To allow comparison of the critical effects of age on concrete and abstract false recognition, Cohen’s *d* (Cohen, 1988) measures of effect size are given for differences in mean false recognition between YAs and OAs for each stimulus type. Effect sizes are also reported for *F*-tests of similarity effects, permitting comparison of the magnitude of perceptual and conceptual similarity effects, using $\eta_{p}^{2}$ (Cohen, 1973). Large effects are defined as *d* of >0.8 and $\eta_{p}^{2}$ of >0.14.

### SIMILARITY RATINGS

The raw ratings data are not included in this report but are available from the first author on request. ANOVAs examined effects of Rater Age (young, older) and Category Type (abstract, concrete) on perceptual and conceptual ratings for each category. Within categories, exemplars were rated as more perceptually similar by YAs than OAs, and concrete items were rated as more perceptually similar than abstract items (Age: *F*_(1,46)_ = 63.34; Category Type: *F*_(1,46)_ = 71.35, *p*s < 0.001). Effects of Age and Category Type interacted (*F*_(1,46)_ = 34.40, *p* < 0.001), such that only abstract items were rated as more perceptually similar by YAs (abstract: *t*_(23)_ = 9.57, *p* < 0.001; concrete: *t*_(23)_ = 0.72, *p* = 0.48). Conceptual ratings did not differ by Rater Age or Category Type (Age: *F*_(1,46)_ = 0.02, *p* = 0.89; Category Type: *F*_(1,46)_ = 0.05, *p* = 0.83; interaction: *F*_(1,46)_ = 0.88, *p* = 0.35), although it should be noted that as the conceptual task differed for concrete and abstract items, these ratings are not directly comparable. Perceptual and conceptual ratings of abstract categories did not correlate reliably in young or older raters (young: *r* = 0.36, *p* = 0.08; older: *r* = 0.23, *p* = 0.29), indicating the scales were indeed measuring distinct stimulus qualities. For concrete categories, perceptual and conceptual ratings were positively correlated in both age groups (young: *r* = 0.66, *p* < 0.001; older: *r* = 0.45, *p* = 0.03).

Across both item types, YAs and OAs showed high, positive correlations for both perceptual and conceptual ratings (perceptual: *r* = 0.83, *p* < 0.001; conceptual: *r* = 0.6, *p* < 0.001). Therefore, to obtain perceptual and conceptual similarity scales which were equally applicable to both age groups, ratings from YAs and OAs were averaged. In the averaged scale, perceptual and conceptual ratings again did not correlate for abstract items (*r* = 0.32, *p* = 0.12), but were positively correlated for concrete items (*r* = 0.69, *p* < 0.001). Concrete items were rated more perceptually similar than abstract items (*M*_concrete_ = 2.89, SD = 0.50; *M*_abstract_ = 3.65, SD = 0.37; *t*_(46)_ = 8.45, *p* < 0.001; lower figures represent greater similarity), while no difference in mean conceptual rating was found between item types (*M*_concrete_ = 2.92, SD = 0.40; *M*_abstract_ = 2.77, SD = 0.25; *t*_(46)_ = 0.22, *p* = 0.83). Proportions of participants assigning the same conceptual label to abstract categories ranged from 0 (each gave a different label) to 0.45 (10/22 gave the same label), with a mean proportion of 0.23 (5/22).

### COGNITIVE TESTS

Cognitive test results for participants in Experiments 1 and 2 are summarized in **Table 1**. In Experiment 1, one OA did not complete the Digit Span Backward task, and WTAR Standard Scores were excluded for five YAs who were non-native speakers of English. YAs and OAs did not differ in years of education, WTAR (Standard Scores), or Digit Span Forward or Backward (education: *t*_(41)_ = 1.10, *p* = 0.28; WTAR: *t*_(41)_ = 1.21, *p* = 0.23; Digit Span Forward: *t*_(46)_ = 1.02, *p* = 0.31; Digit Span Backward: *t*_(46)_ = 0.82, *p* = 0.42). YAs outperformed OAs on the Digit Symbol task (*t*_(46)_ = 5.56, *p* < 0.001) as expected. Chi-squared test of independence confirmed that the sex distribution did not differ between age groups [χ^2^(1, *N* = 48) = 0.08, *p* = 0.77].

In Experiment 2, one OA was unable to complete the Digit Symbol test due to an injury, and one YA was excluded from this test due to failure to follow procedure. WTAR scores were disregarded for 10 YAs who were not native speakers of English. The proportion of females was higher in the young group [Chi-squared test of independence: χ^2^(1, *N* = 52) = 4.28, *p* < 0.05]. However, rates of veridical recognition, lure false recognition, or lure correct rejections did not differ by sex for either abstract or concrete stimuli, suggesting bias of results was unlikely. YAs again scored more highly on the Digit Symbol task (*t*_(48)_ = 5.28, *p* < 0.001). No age differences were observed in years of education, WTAR (Standard Score), or Digit Span Forward or Backward (education: *t*_(50)_ = 0.06, *p* = 0.95; WTAR: *t*_(40)_ = 0.71, *p* = 0.48; Digit Span Forward: *t*_(50)_ = 0.74, *p* = 0.46; Digit Span Backward: *t*_(50)_ = 1.1, *p* = 0.29).

Comparing samples for the two experiments, 2 (Age) × 2 (Experiment) ANOVAs showed an effect of Experiment for Digit Symbol Backward, with participants in Experiment 1 outperforming those in Experiment 2 (*F*_(1,95)_ = 6.77, *p* = 0.011). No further differences between samples were observed in age, years of education, sex or cognitive test performance, and there were no interactions of Experiment × Age (max *F* = 1.82; **Table 1**).

In both experiments, memory performance results (including effects of similarity) were equivalent when non-native speakers of English were excluded from analyses, and so only results from the full samples are reported.

### EXPERIMENT 1 – MEMORY PERFORMANCE

#### Baseline false recognition of novel items

We examined effects of Stimulus Type (abstract, concrete) and Age (young, old) on baseline novel false recognition. More abstract items were falsely recognized than concrete (*F*_(1,46)_ = 52.41, *p* < 0.001; *M*_abstract_ = 0.12, SD = 0.17; *M*_concrete_ = 0.03, SD = 0.04), but this effect did not differ by Age (*F*_(1,46)_ = 0.06, *p* = 0.82), and no overall effect of Age was observed (*F* < 1). Findings did not differ when analyses were restricted to novel items falsely recognized with high confidence, (Stimulus Type: *F*_(1,46)_ = 12.53, *p* = 0.001; Age: *F*_(1,46)_ = 0.32, *p* = 0.57; interaction: *F*_(1,46)_ = 0.56, *p* = 0.46; *M*_abstract_ = 0.04, SD = 0.07; *M*_concrete_ = 0.01, SD = 0.03).

#### Corrected false recognition of lures

**FIGURE 2:**
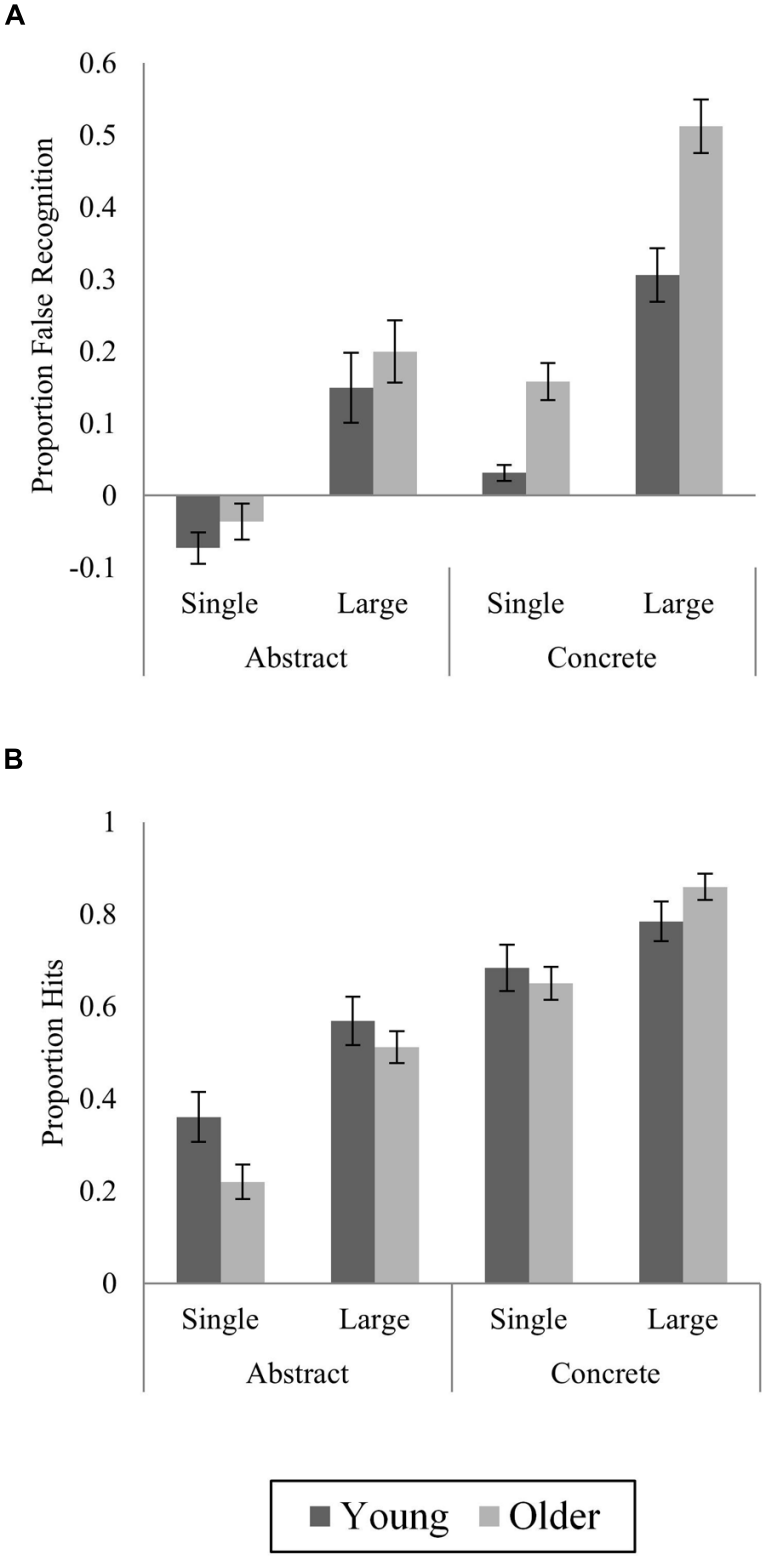
**Novel-corrected proportions of **(A)** lure false recognition (“Old” responses to lures), and **(B)** hits (“Old” responses to studied items) by age, stimulus type, and category size at study in Experiment 1.** Means ± SE.

Following Koutstaal et al. (2003), lure false recognition was corrected for baseline false recognition of novel items. Proportions of false recognition of abstract and concrete novel items were subtracted from proportions of false recognition of abstract and concrete lures, respectively. Corrected false recognition is shown in **Figure 2A**. ANOVA with factors of Age (young, older), Category Size at study (single, large) and Stimulus Type (abstract, concrete) revealed significant main effects of all three variables (Age: *F*_(1,46)_ = 13.88, *p* = 0.001; Category Size: *F*_(1,46)_ = 123.41, *p* < 0.001; Stimulus Type: *F*_(1,46)_ = 81.48, *p* < 0.001), reflecting greater false recognition among OAs, for large category items, and for concrete items. Crucially, the predicted Age × Stimulus Type interaction was significant (*F*_(1,46)_ = 8.41, *p* = 0.006), with greater false recognition among OAs compared to YAs for concrete items, but no difference for abstract items (concrete: *t*_(46)_ = 5.29, *p* < 0.001, *d* = 1.53; abstract: *t*_(46)_ = 1.12, *p* = 0.27, *d* = 0.32). The effect of Category Size interacted with Stimulus Type (*F*_(1,46)_ = 4.84, *p* = 0.03), with a larger effect of Category Size (greater false recognition of large category lures) for concrete items, but neither this interaction or the main effect of Category Size differed by Age (Category Size × Stimulus Type × Age: *F*_(1,46)_ = 0.73, *p* = 0.4; Category Size × Age: *F*_(1,46)_ = 0.92, *p* = 0.34). Following Koutstaal et al. (2003), we also assessed novel-corrected false recognition of single and large category abstract items alone, to determine whether age-related differences were truly unique to concrete items. As predicted by the semantic categorization account, no age differences were observed (single: *t*_(46)_ = 1.1, *p* = 0.28; large: *t*_(46)_ = 0.78, *p* = 0.44).

Restricting analyses to highly confident false recognition, corrected for highly confident novel false recognition (**Table 2**), similar results were observed. OAs showed greater confident false recognition than YAs (*F*_(1,46)_ = 25.94, *p* < 0.001), concrete lures were more often confidently falsely recognized than abstract lures (*F*_(1,46)_ = 66.09, *p* < 0.001), and large category lures attracted more highly confident false recognition responses than single-item categories (*F*_(1,46)_ = 92.14, *p* < 0.001). A Stimulus Type × Category Size interaction (*F*_(1,46)_ = 13.71, *p* = 0.001) reflected a greater effect of Category Size for concrete items. A greater effect of Category Size was also observed in OAs compared to YAs (*F*_(1,46)_ = 11.34, *p* = 0.002). The predicted Stimulus Type × Age interaction was again observed (*F*_(1,46)_ = 7.34, *p* = 0.009; Category Size × Stimulus Type × Age: *F*_(1,46)_ = 0.55, *p* = 0.46) however, unlike in the overall analysis OAs showed greater false recognition for abstract (*t*_(46)_ = 2.93, *p* = 0.005, *d* = 0.85) as well as concrete items (*t*_(46)_ = 5.28, *p* < 0.001, *d* = 1.52) with age effects larger for concrete items. Planned tests of age differences among abstract items alone revealed greater highly confident false recognition in OAs of large but not single category abstract lures (single: *t*_(46)_ = 1.14, *p* = 0.26; large: *t*_(46)_ = 2.66, *p* = 0.01).

**Table 2:** Mean proportions (SD) of novel-corrected highly confident lure false recognition and novel-corrected highly confident hits to studied items in Experiment 1.

	Abstract				Concrete			
	Single		Large		Single		Large	
	YA	OA	YA	OA	YA	OA	YA	OA
Lure FR	–0.02 (0.05)	–0.003 (0.05)	0.05 (0.10)	0.16 (0.19)	0.02 (0.04)	0.09 (0.12)	0.16 (0.11)	0.37 (0.16)
Hits	0.35 (0.23)	0.20 (0.18)	0.46 (0.22)	0.44 (0.24)	0.65 (0.26)	0.54 (0.23)	0.65 (0.26)	0.75 (0.26)

*FR = false recognition.*

#### Corrected veridical recognition

As for false recognition, proportions of hits (correctly identified old items) were corrected for baseline novel false recognition (**Figure 2B**) equivalent to Snodgrass and Corwin’s (1988) *P*_r_ measure. Proportion of novel-corrected hits did not vary by age (*F*_(1,46)_ = 0.75, *p* = 0.39), but effects of Stimulus Type and Category Size were greater in OAs (Stimulus Type × Age: *F*_(1,46)_ = 4.39, *p* = 0.04; Category Size × Age: *F*_(1,46)_ = 4.41, *p* = 0.04). Main effects of Category Size (*F*_(1,46)_ = 79.21, *p* < 0.001) and Stimulus Type (*F*_(1,46)_ = 132.46, *p* < 0.001) were modified by a Stimulus Type × Category Size interaction (*F*_(1,46)_ = 7.27, *p* = 0.01), reflecting a greater increment in hit rate with larger Category Size for abstract (25%) compared to concrete (15.5%) items. This interaction did not however, differ by Age (3-way interaction: *F*_(1,46)_ = 0.12, *p* = 0.73).

For highly confident hits corrected for highly confident novel false recognition (**Table 2**), the Stimulus Type × Age interaction was not reliable (*F*_(1,46)_ = 2.41, *p* = 0.13), nor was the Category Size × Stimulus Type interaction (*F*_(1,46)_ = 2.47, *p* = 0.12). However, the remaining effects were equivalent to those for total hits (Age: *F*_(1,46)_ = 0.72, *p* = 0.4; Stimulus Type: *F*_(1,46)_ = 119.64, *p* < 0.001; Category Size: *F*_(1,46)_ = 38.85, *p* < 0.001; Category Size × Age: *F*_(1,46)_ = 13.18, *p* = 0.001).

#### Effects of stimulus perceptual and conceptual similarity

**FIGURE 3:**
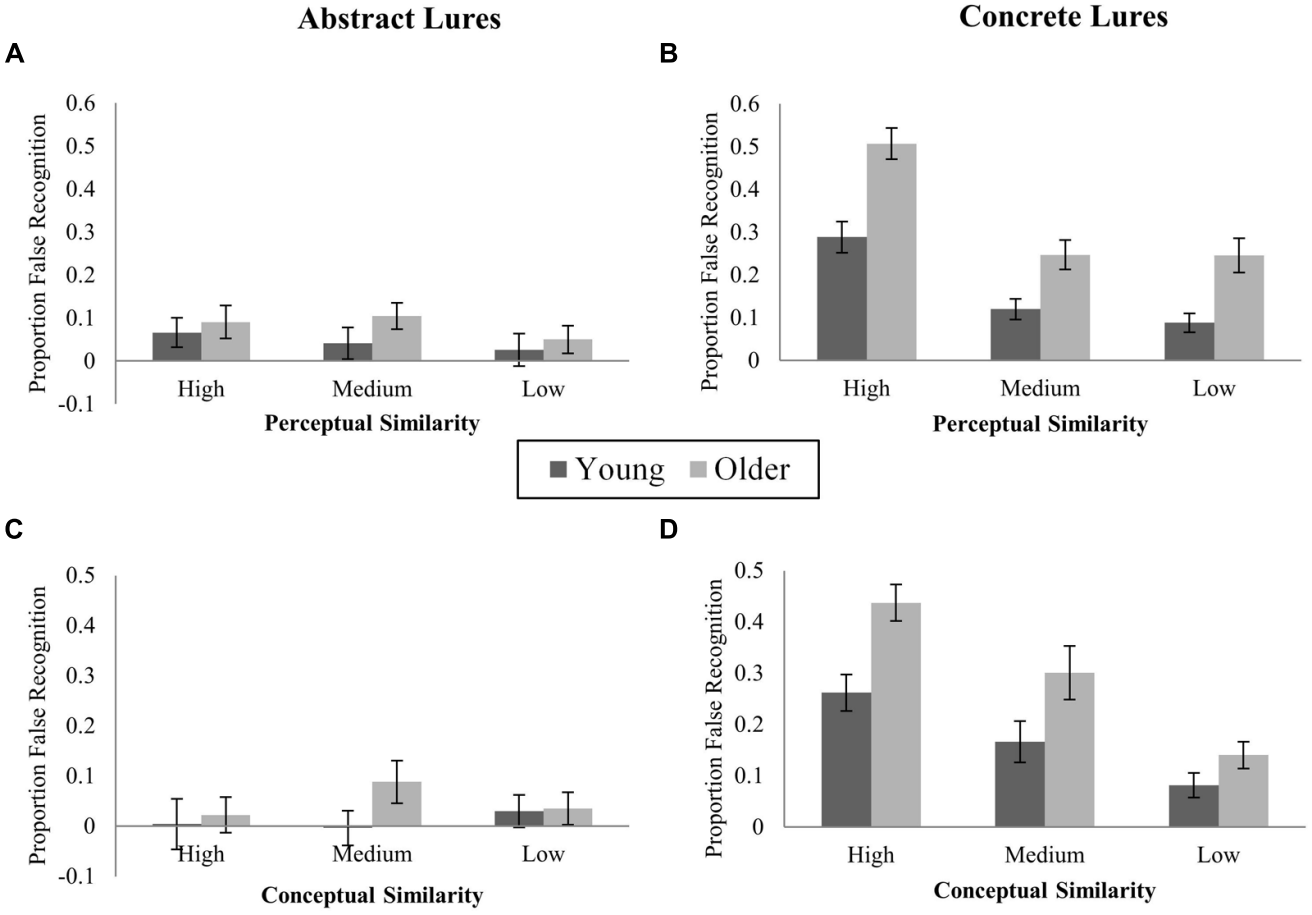
**Proportions of novel-corrected false recognition (“Old” responses to lures) to high, medium, and low similarity lures in Experiment 1. (A)** Abstract lures; high, medium, and low perceptual similarity; **(B)** Concrete lures; high, medium, and low perceptual similarity; **(C)** Abstract lures; high, medium, and low conceptual similarity; **(D)** Concrete lures; high, medium, and low conceptual similarity.

Novel-corrected false recognition of abstract and concrete images according to within-category conceptual and perceptual similarity is illustrated in **Figure 3**. For each rating (perceptual and conceptual), ANOVAs examined effects of Age (young, older) and Similarity (high, medium, low) on novel-corrected false recognition of abstract and concrete lures, combining across both category sizes. As the perceptual similarity ratings task was equal for abstract and concrete stimuli, Stimulus Type was included as a factor in perceptual similarity analyses. However, as conceptual ratings tasks differed for abstract and concrete images, separate ANOVAs were conducted for each stimulus type. Planned comparisons of age effects at each level of similarity were conducted to test pattern separation predictions in all cases where there were significant effects of similarity.

***Perceptual similarity.*** False recognition rates differed according to within-category Perceptual Similarity (*F*_(2,92)_ = 32.67, *p* < 0.001, $\eta_{p}^{2}$ = 0.42), with highly perceptually similar lures attracting more false recognition responses than the most perceptually distinctive lures. This effect was modified by Stimulus Type (*F*_(2,92)_ = 14.92, *p* < 0.001) and was reliable for concrete lures only (concrete: *F*_(2,92)_ = 39.08, *p* < 0.001, $\eta_{p}^{2}$ = 0.46; abstract: *F*_(2,92)_ = 1.39, *p* = 0.26, $\eta_{p}^{2}$ = 0.03). Across both age groups, false recognition was greater for high vs. both medium and low similarity concrete lures, but did not differ between medium and low similarity concrete lures (high vs. medium: *t*_(47)_ = 7.28, *p* < 0.001; high vs. low: *t*_(47)_ = 8.16, *p* < 0.001; medium vs. low: *t*_(47)_ = 0.56, *p* = 0.58, adjusted α = 0.017; Perceptual Similarity × Age: *F*_(2,92)_ = 1.28, *p* = 0.28; 3-way interaction: *F*_(2,92)_ = 0.43, *p* = 0.65). In the main ANOVA, the interaction of Stimulus Type × Similarity did not differ by Age (3-way interaction: *F*_(2,92)_ = 0.43, *p* = 0.65).

Predictions from pattern separation models that OAs requires greater change in input (i.e., less similarity) to support successful discrimination were tested among concrete items (for which a significant effect of Perceptual Similarity was observed) via planned contrasts of group differences at each level of similarity. OAs were more likely than YAs to falsely recognize concrete lures at all levels of Perceptual Similarity (high: *t*_(46)_ = 4.24, *p* < 0.001; medium: *t*_(46)_ = 3.01, *p* = 0.004; low: *t*_(46)_ = 3.43, *p* = 0.001, adjusted α = 0.017).

***Conceptual similarity.*** For abstract items, no reliable effect of Conceptual Similarity was observed (*F*_(2,92)_ = 0.48, *p* = 0.62, $\eta_{p}^{2}$ = 0.01; Age × Similarity: *F*_(2,92)_ = 1.22, *p* = 0.30). For concrete items, false recognition varied according to Conceptual Similarity (*F*_(2,92)_ = 28.44, *p* < 0.001, $\eta_{p}^{2}$ = 0.38). Lures from highly conceptually similar categories were falsely recognized more often than medium or low similarity lures, and more medium than low similarity lures were falsely recognized (high vs. medium: *t*_(47)_ = 3.45, *p* = 0.001; high vs. low: *t*_(47)_ = 8.63, *p* < 0.001; medium vs. low: *t*_(47)_ = 3.61; *p* = 0.001; adjusted α = 0.017). Although OAs showed a numerically greater increment in false recognition from low to high similarity (OA: 29.6%, YA: 18%), the Similarity × Age interaction was not reliable (*F*_(2,92)_ = 1.74, *p* = 0.18). For concrete items, planned contrasts revealed greater false recognition in OAs than YAs for highly conceptually similar lures (*t*_(46)_ = 3.5, *p* = 0.001), but not medium or low similarity lures (medium: *t*_(46)_ = 2.05, *p* = 0.046; low: *t*_(46)_ = 1.67, *p* = 0.1; adjusted α = 0.017).

### EXPERIMENT 2

#### Memory performance

**FIGURE 4:**
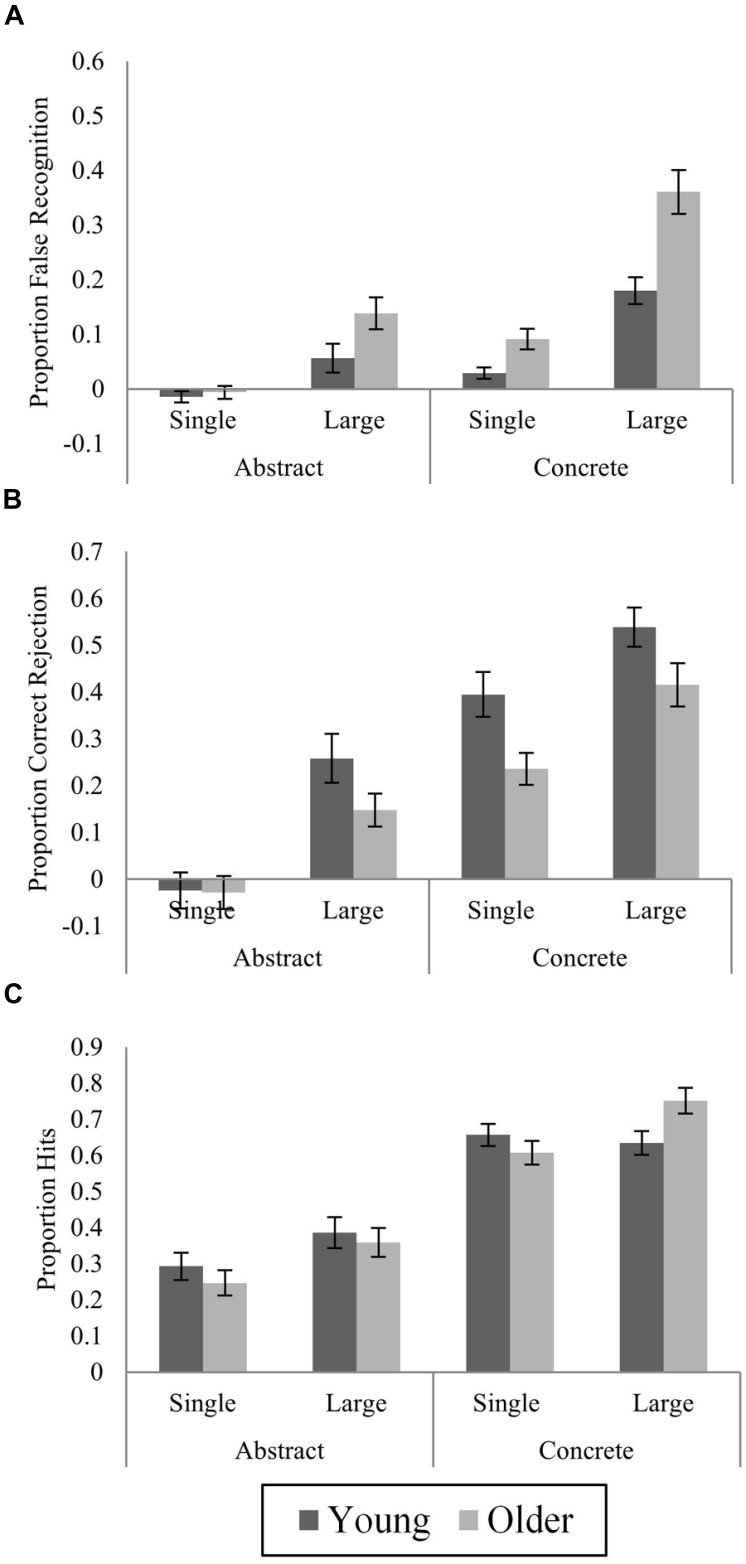
**Proportions of novel-corrected **(A)** lure false recognition (“Old” responses to lures), **(B)** lure correct rejection (“Similar” responses to lures) and **(C)** hits (“Old” responses to studied items) by age, stimulus type, and category size at study in Experiment 2.** All proportions are corrected for proportions of “old” (for false recognition and hits) or “similar” (for correct rejection) responses to novel items. Mean ± SE.

With the added “similar” response option in Experiment 2, there were nine possible response outcomes. Studied items could be correctly recognized (hits), judged “similar,” or missed (judged “new”). Lures could be falsely recognized as “old,” correctly rejected as “similar,” or incorrectly judged “new.” Novel items could be falsely recognized as “old” (novel false recognition); incorrectly judged “similar,” or correctly rejected as “new.” Raw proportions of responses in each of these categories are displayed in **Table 3**. Analyses focused on novel-corrected hits, and lure false recognition and correct rejection, illustrated in **Figure 4**.

**Table 3:** Proportions of raw “Old,” “Similar” and “New” responses to Studied, Lure and Novel items, collapsed across single and large category conditions.

		Abstract		Concrete	
		YA	OA	YA	OA
Studied	“Old”	0.38 (0.17)	0.35 (0.20)	0.66 (0.11)	0.71 (0.15)
	“Similar”	0.35 (0.13)	0.41 (0.19)	0.19 (0.08)	0.17 (0.11)
	“New”	0.20 (0.14)	0.19 (0.08)	0.08 (0.05)	0.08 (0.08)
Lure	“Old”	0.06 (0.09)	0.12 (0.11)	0.12 (0.08)	0.25 (0.15)
	“Similar”	0.43 (0.18)	0.42 (0.14)	0.53 (0.21)	0.37 (0.18)
	“New”	0.44 (0.19)	0.41 (0.17)	0.30 (0.19)	0.33 (0.13)
Novel	“Old”	0.04 (0.06)	0.05 (0.06)	0.02 (0.04)	0.03 (0.07)
	“Similar”	0.32 (0.22)	0.36 (0.18)	0.06 (0.10)	0.04 (0.06)
	“New”	0.59 (0.24)	0.54 (0.21)	0.89 (0.16)	0.91 (0.14)

*Mean (standard deviation).*

***Baseline false recognition of novel items.*** Analysis of variance examining effects of Stimulus Type and Age on proportions of baseline false recognition (**Table 3**) showed that this was more likely for abstract than concrete novel items (*F*_(1,50)_ = 8.78, *p* = 0.005). There was no main effect of Age (*F*_(1,50)_ = 0.67, *p* = 0.42), and no Stimulus Type × Age interaction (*F*_(1,50)_ < 0.001, *p* = 1). Among highly confident responses, rates of novel false recognition no longer differed according to Stimulus Type (*F*_(1,50)_ = 1.12, *p* = 0.30), but there remained no effects involving Age (Age: *F*_(1,50)_ = 0.48, *p* = 0.49; Stimulus Type × Age: *F*_(1,50)_ = 0.50, *p* = 0.48).

***Corrected false recognition of lures.*** Proportions of abstract and concrete lure false recognition were corrected for baseline false recognition of novel abstract and concrete items as in Experiment 1, and are displayed in **Figure 4A**. For lure false recognition, ANOVA with factors of Stimulus Type, Category Size, and Age revealed significant main effects of each (Stimulus Type: *F*_(1,50)_ = 55.07; Category Size: *F*_(1,50)_ = 94.04; Age: *F*_(1,50)_ = 17.93, *p*s < 0.001), with greater false recognition of concrete lures relative to abstract, of large category items vs. single, and in OAs. As expected, the effect of Stimulus Type was modulated by Age (*F*_(1,50)_ = 5.49, *p* = 0.023). There was a clear age difference for concrete items (*t*_(50)_ = 4.25, *p* < 0.001, *d* = 1.18), with OAs 12.2% more likely to falsely recognize concrete lures, and a smaller but significant age difference for abstract items (4.5%; *t*_(50)_ = 2.02, *p* = 0.049, *d* = 0.56). The effect of Category Size also differed by Age (*F*_(1,50)_ = 8.64, *p* = 0.005). In both age groups, large category lures attracted more false recognition responses than single category lures (YAs: *t*_(25)_ = 5.65; OAs: *t*_(25)_ = 8.95, *p*s < 0.001), but the effect of Category Size was larger in OAs. The effect of Stimulus Type was modulated by Category Size (*F*_(1,50)_ = 13.24, *p* = 0.001), with a larger effect of Category Size for concrete items. No three-way interaction was observed (*F*_(1,50)_ = 0.63, *p* = 0.43). Age differences among abstract lures alone were again tested. OAs were more likely than YAs to falsely recognize large but not single category abstract lures (single: *t*_(50)_ = 0.51, *p* = 0.61; large: *t*_(50)_ = 2.07, *p* = 0.043).

Similar effects were observed for novel-corrected highly confident false recognition (**Table 4**), i.e., main effects of Stimulus Type, Category Size, and Age (Stimulus Type: *F*_(1,50)_ = 47.16; Category Size: *F*_(1,50)_ = 73.89; Age: *F*_(1,50)_ = 18.01, *p*s < 0.001), and interactions of Category Size with Stimulus Type and Age (Category Size × Stimulus Type: *F*_(1,50)_ = 12.51, *p* = 0.001; Category Size × Age: *F*_(1,50)_ = 11.04, *p* = 0.002; Category Size × Stimulus Type × Age: *F*_(1,50)_ = 0.63, *p* = 0.43). As in Experiment 1, the effect of Stimulus Type was modulated by Age (*F*_(1,50)_ = 8.07, *p* = 0.007), but the age difference in false recognition was now reliable for concrete items only (concrete: *t*_(50)_ = 4.30, *p* < 0.001, $\eta_{p}^{2}$ = 1.19; abstract: *t*_(50)_ = 1.93, *p* = 0.059, $\eta_{p}^{2}$ = 0.54). However, in planned analysis of abstract lures, OAs again showed higher confident false recognition of large but not single abstract lures (single: *t*_(50)_ = 0.50, *p* = 0.62; large: *t*_(50)_ = 2.28, *p* = 0.027).

**Table 4:** Mean proportions (SD) of novel-corrected highly confident lure false recognition, novel-corrected highly confident correct rejections and novel-corrected highly confident hits in Experiment 2.

	Abstract				Concrete			
	Single		Large		Single		Large	
	YA	OA	YA	OA	YA	OA	YA	OA
Lure FR	–0.002 (0.04)	–0.02 (0.03)	0.04 (0.09)	0.12 (0.15)	0.03 (0.04)	0.09 (0.10)	0.14 (0.11)	0.33 (0.21)
Lure CR	0.01 (0.12)	0.01 (0.11)	0.25 (0.23)	0.12 (0.14)	0.37 (0.24)	0.14 (0.12)	0.47 (0.22)	0.34 (0.24)
Hits	0.27 (0.19)	0.24 (0.17)	0.33 (0.20)	0.31 (0.19)	0.64 (0.15)	0.53 (0.19)	0.60 (0.18)	0.74 (0.18)

*FR = false recognition (lures judged “old”); CR = correct rejection (lures judged “similar”).*

***Correct rejection of lures.*** Proportions of lure correct rejections were corrected by subtracting proportions of “similar” responses to novel items of the same stimulus type (equal to Stark et al.’s (2013) behavioral pattern separation score), and are displayed in **Figure 4B**. Main effects of Stimulus Type, Category Size and Age were observed (Stimulus Type: *F*_(1,50)_ = 108.21, *p* < 0.001; Category Size: *F*_(1,50)_ = 79.31, *p* < 0.001; Age: *F*_(1,50)_ = 5.56, *p* = 0.022), with YAs 14.1% more likely than OAs to correctly reject concrete lures, and only 5.7% more likely to correctly reject abstract lures, but the predicted Age × Stimulus Type interaction was non-significant (*F*_(1,50)_ = 1.98, *p* = 0.17), and did not vary by Category Size (3-way interaction: *F*_(1,50)_ = 3.14, *p* = 0.08), suggesting OAs were impaired in correct rejection of both abstract and concrete lures. Planned contrasts for abstract items alone did not show reliable age-related differences for correct rejection of either single or large category abstract lures (single: *t*_(50)_ = 0.09, *p* = 0.93; large: *t*_(50)_ = 1.75, *p* = 0.09).

Rates of novel-corrected highly confident correct rejection are shown in **Table 4**. A three-way Stimulus Type × Category Size × Age interaction was observed (*F*_(1,50)_ = 11.21, *p* = 0.002), as well as the predicted Stimulus Type × Age interaction (*F*_(1,50)_ = 4.88, *p* = 0.03). Lure correct rejection was more likely in YAs than OAs for both large and single category concrete lures (large: *t*_(50)_ = 2.07, *p* = 0.04; single: *t*_(50)_ = 4.31, *p* < 0.001), and for large category abstract lures (*t*_(50)_ = 2.67, *p* = 0.01), but not for single category abstract lures (*t*_(50)_ = 0.05, *p* = 0.96).

***Corrected veridical recognition.*** Novel-corrected hits to studied items are shown in **Figure 4C**. Concrete images were correctly recognized more often than abstract (*F*_(1,50)_ = 186.26, *p* < 0.001), and more large than single category items were recognized (*F*_(1,50)_ = 14.19, *p* < 0.001), though there was no main effect of Age (*F*_(1,50)_ = 0.002, *p* = 0.96). A marginally significant 3-way interaction (*F*_(1,50)_ = 4.0, *p* = 0.05) reflected presence of a Category Size × Age interaction for concrete (*F*_(1,50)_ = 8.66, *p* = 0.005) but not abstract items (*F*_(1,50)_ = 0.11, *p* = 0.74). OAs were more likely than YAs to recognize large category concrete items (*t*_(50)_ = 2.43, *p* = 0.019), but no age difference was present for single-items (*t*_(50)_ = 1.10, *p* = 0.28).

Similar effects were found for novel-corrected highly confident responses, shown in **Table 4** (Age: *F*_(1,50)_ = 0.04, *p* = 0.84; Stimulus Type: *F*_(1,50)_ = 198.45, *p* < 0.001; Category Size: *F*_(1,50)_ = 14.58, *p* < 0.001; Category Size × Age: *F*_(1,50)_ = 11.06, *p* = 0.002). A three-way interaction (*F*_(1,50)_ = 8.64, *p* = 0.005) again reflected a Category Size × Age interaction among concrete items only (concrete: *F*_(1,50)_ = 18.60, *p* < 0.001; abstract: *F*_(1,50)_ = 0.13, *p* = 0.72), with OAs more likely than YAs to recognize large category concrete items (*t*_(50)_ = 2.72, *p* = 0.009).

#### Effects of stimulus perceptual and conceptual similarity

**FIGURE 5:**
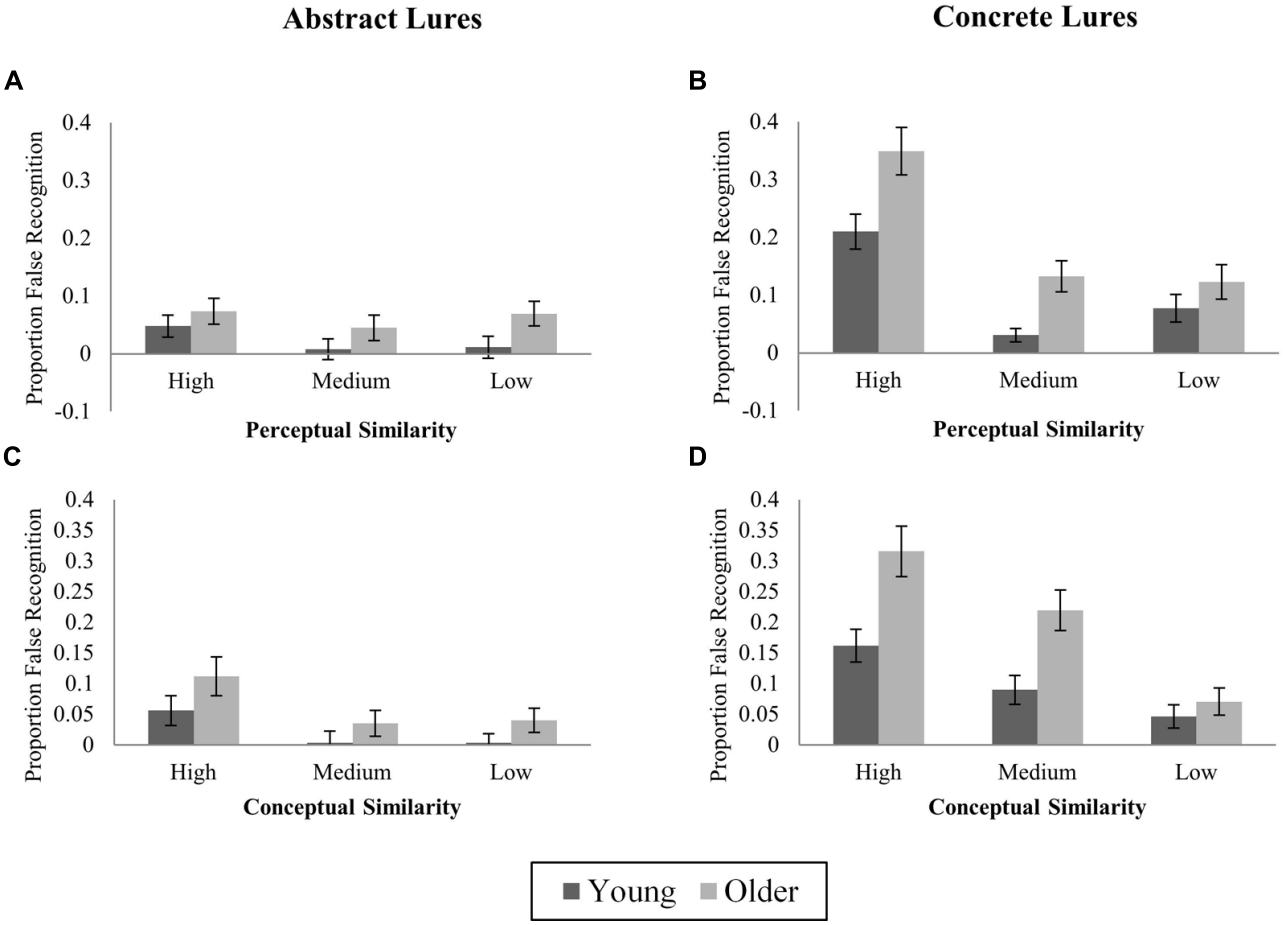
**Proportions of novel-corrected false recognition (“Old” responses to lures) to high, medium, and low similarity lures. (A)** Abstract lures, perceptual similarity; **(B)** Concrete lures, perceptual similarity; **(C)** Abstract lures, conceptual similarity; **(D)** Concrete lures, conceptual similarity. Mean ± SE.

**FIGURE 6:**
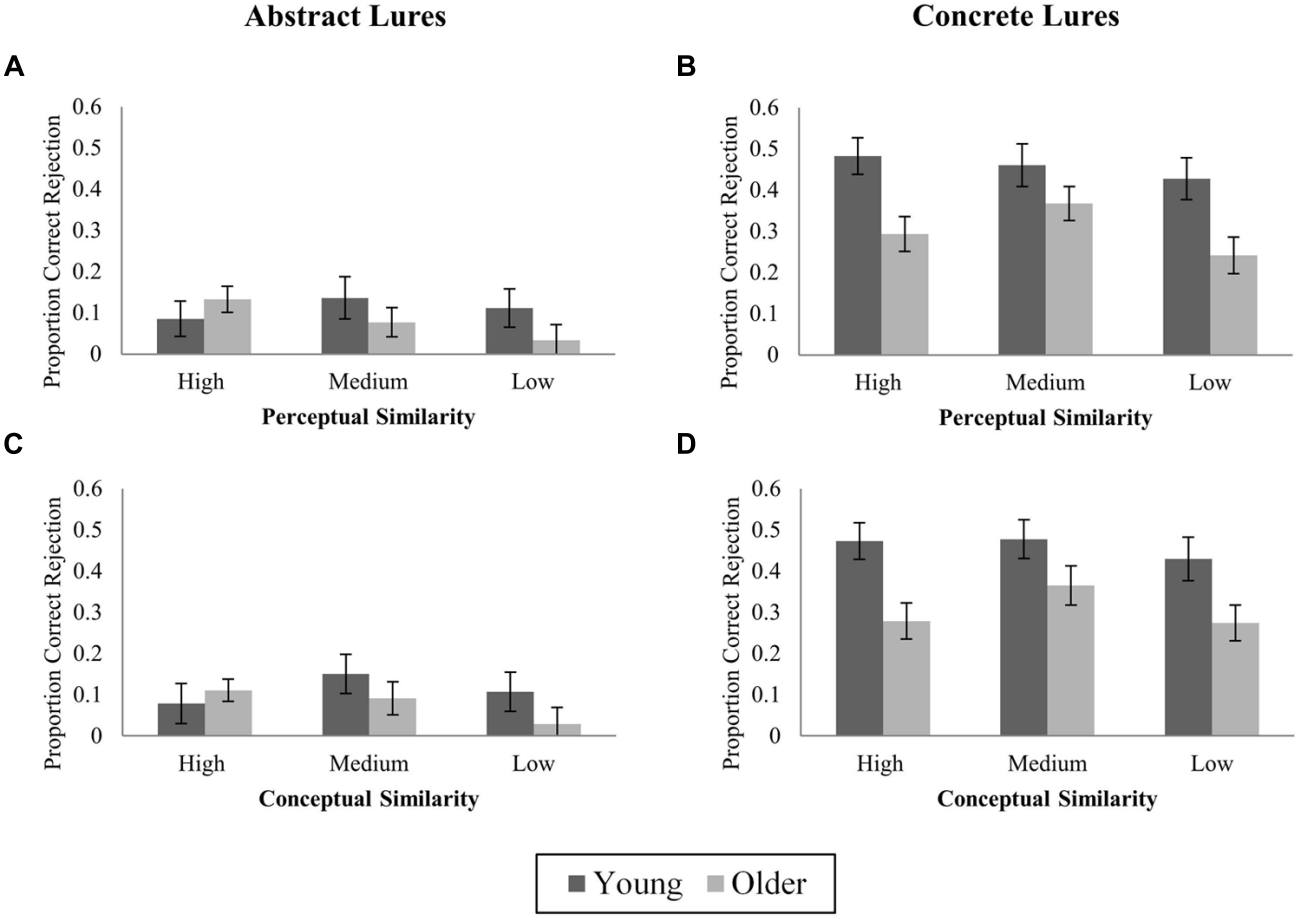
**Proportions of novel-corrected correct rejection (“Similar” responses to lures) to high, medium, and low similarity lures. (A)** Abstract lures, perceptual similarity; **(B)** Concrete lures, perceptual similarity; **(C)** Abstract lures, conceptual similarity; **(D)** Concrete lures, conceptual similarity. Mean ± SE.

Proportions of novel-corrected lure false recognition and correct rejection according to input similarity are shown in **Figures 5 and 6**, respectively. Analyses followed the same strategy as in Experiment 1, but examined lure correct rejections as well as false recognition.

***Perceptual similarity.*** Analysis of variance revealed a significant effect of rated Perceptual Similarity on false recognition (*F*_(2,100)_ = 39.81, *p* < 0.001, $\eta_{p}^{2}$ = 0.44) modified by an interaction with Stimulus Type (*F*_(2,100)_ = 19.39, *p* < 0.001). The effect of Perceptual Similarity was significant for concrete items only (concrete: *F*_(2,100)_ = 42.16, *p* < 0.001, $\eta_{p}^{2}$ = 0.46; abstract: *F*_(2,100)_ = 2.15, *p* = 0.12, $\eta_{p}^{2}$ = 0.04). Highly perceptually similar concrete lures were more often falsely recognized than medium or low similarity lures, but false recognition of medium and low similarity lures did not differ (high vs. medium: *t*_(51)_ = 8.46; high vs. low: *t*_(51)_ = 7.22; *p*s < 0.001; medium vs. low: *t*_(51)_ = 0.77, *p* = 0.45, adjusted α = 0.017). The effect of Perceptual Similarity did not vary with Age (*F*_(2,100)_ = 1.98, *p* = 0.14; 3-way interaction, *F*_(2,100)_ = 2.27, *p* = 0.11). As in Experiment 1, planned contrasts examined age-related differences at each level of Perceptual Similarity for concrete items, for which the overall effect of similarity was reliable. OAs were more likely to falsely recognize high and medium perceptual similarity concrete lures, but not the most distinctive lures (high: *t*_(50)_ = 2.74, *p* = 0.008; medium: *t*_(50)_ = 3.49, *p* = 0.001; low: *t*_(50)_ = 1.18, *p* = 0.24, adjusted α = 0.017).

For lure correct rejections, we observed a main effect of Perceptual Similarity and a 3-way interaction (Perceptual Similarity: *F*_(1,50)_ = 3.17, *p* = 0.046; Perceptual Similarity × Stimulus Type × Age: *F*_(2,100)_ = 3.09, *p* = 0.05; Similarity × Stimulus Type, *F*_(2,100)_ = 0.60, *p* = 0.55). *Post hoc* tests showed an Age × Stimulus Type interaction for highly perceptually similar lures only (high: *F*_(1,50)_ = 10.53, *p* = 0.002; medium: *F*_(1,50)_ = 0.18, *p* = 0.67; low: *F*_(1,50)_ = 2.10, *p* = 0.15). This reflected higher correct rejection among YAs of highly similar concrete lures, but no age difference for highly similar abstract lures (concrete: *t*_(50)_ = 3.09, *p* = 0.003; abstract: *t*_(50)_ = 0.90, *p* = 0.37). Planned tests of age differences at each level of similarity were conducted for both abstract and concrete lures. Abstract lures did not show reliable age differences at any level of similarity (high: *t*_(50)_ = 0.90, *p* = 0.37; medium: *t*_(50)_ = 0.96, *p* = 0.34; low: *t*_(50)_ = 1.30, *p* = 0.20; α = 0.017). For concrete lures, YAs showed greater correct rejection than OAs of the most perceptually similar and most perceptually distinctive lures, but no difference for medium similarity lures (high: *t*_(50)_ = 3.09, *p* = 0.003; medium: *t*_(50)_ = 1.40, *p* = 0.17; low: *t*_(50)_ = 2.77, *p* = 0.008; adjusted α = 0.017).

***Conceptual similarity.*** Conceptual Similarity had a significant effect on novel-corrected false recognition of abstract lures (*F*_(2,100)_ = 6.75, *p* = 0.002, $\eta_{p}^{2}$ = 0.12). Highly conceptually similar lures were falsely recognized more often than medium or low similarity lures (high vs. medium: *t*_(51)_ = 2.66, *p* = 0.01; high vs. low: *t*_(51)_ = 3.01, *p* = 0.004). The similarity effect did not differ by age (*F*_(2,100)_ = 0.20, *p* = 0.82), and there were no age differences at any level of similarity (high: *t*_(50)_ = 1.40, *p* = 0.17; medium: *t*_(50)_ = 1.12, *p* = 0.27; low: *t*_(51)_ = 1.50, *p* = 0.14, adjusted α = 0.017).

False recognition of concrete lures also differed according to Conceptual Similarity (*F*_(2,100)_ = 26.41, *p* < 0.001, $\eta_{p}^{2}$ = 0.35), with the most conceptually similar lures attracting the highest levels of false recognition. Crucially, Age interacted with Conceptual Similarity (*F*_(2,100)_ = 3.87, *p* = 0.024), as there was a significantly greater drop in false recognition from high to low similarity lures among OAs (24.5%) compared to YAs (11.5%; *t*_(50)_ = 2.98, *p* = 0.004; **Figure 5**). OAs showed higher false recognition than YAs for high and medium, but not low similarity lures (high: *t*_(50)_ = 3.13, *p* = 0.003; medium: *t*_(50)_ = 3.17, *p* = 0.003; low: *t*_(50)_ = 0.82, *p* = 0.42; adjusted α = 0.017).

Conceptual Similarity did not reliably affect correct rejection of either abstract or concrete lures (abstract: *F*_(2,100)_ = 1.39, *p* = 0.25; concrete: *F*_(2,100)_ = 2.12, *p* = 0.13), and did not interact with Age for either Stimulus Type (abstract: *F*_(2,100)_ = 1.73, *p* = 0.18; concrete: *F*_(2,100)_ = 0.71, *p* = 0.50).

### COMPARISON OF FALSE RECOGNITION IN EXPERIMENTS 1 AND 2

We conducted combined analyses of false recognition in Experiments 1 and 2 in order to assess whether addition of a “similar” response option altered false recognition rates, and whether age effects on abstract false recognition were robust across all participants. ANOVAs examining effects of Stimulus Type, Category Size, and Age in addition to a between-subjects factor of Experiments (1, 2) were conducted for total and for highly confident false recognition. Only significant effects involving Experiment, or Stimulus Type and Age are reported, and age effects for abstract items alone.

Corrected false recognition was lower in Experiment 2 for concrete items only (Experiment: *F*_(1,96)_ = 9.26, *p* = 0.003; Experiment × Stimulus Type: *F*_(1,96)_ = 6.93, *p* = 0.01; abstract: *t*_(98)_ = 0.75, *p* = 0.45; concrete: *t*_(98)_ = 3.38, *p* = 0.001). Effects of Experiment also differed by Category Size (*F*_(1,96)_ = 15.18, *p* < 0.001): false recognition was lower for large category lures in Experiment 2 than in Experiment 1, but Experiment had no effect on false recognition of single lures (large: *t*_(98)_ = 3.67, *p* < 0.001; single: *t*_(98)_ = 0.12, *p* = 0.90). None of these effects differed by Age (Experiment × Age: *F*_(1,96)_ = 0.41, *p* = 0.52; Experiment × Stimulus × Age: *F*_(1,96)_ = 0.76, *p* = 0.39; Experiment × Category Size × Age: *F*_(1,96)_ = 0.72, *p* = 0.40). For highly confident false recognition, no effects involving Experiment were significant (max *F* = 2.36).

The three-way interactions of Stimulus Type × Category Size × Age were not significant across experiments for either total (*F*_(1,96)_ = 1.37, *p* = 0.25) or highly confident corrected false recognition (*F*_(1,96)_ = 1.05, *p* = 0.31). Across experiments, the predicted Stimulus Type × Age interaction was reliable for both total and highly confident corrected false recognition (total: *F*_(1,96)_ = 14.14; confident: *F*_(1,96)_ = 15.23, *p*s < 0.001). Although larger age effects were observed for concrete lures (total: *t*_(98)_ = 6.27, *p* < 0.001, *d* = 1.25; confident: *t*_(98)_ = 6.74, *p* < 0.001, *d* = 1.35), age effects were also significant for abstract lures (total: *t*_(98)_ = 2.03, *p* = 0.045, *d* = 0.41; confident: *t*_(98)_ = 3.48, *p* = 0.001, *d* = 0.70). Stimulus Type × Age interactions did not differ by Experiment (total: *F*_(1,96)_ = 0.76, *p* = 0.39; confident: *F*_(1,96)_ = 0.08, *p* = 0.78).

Consistent with findings from each experiment, age differences in abstract false recognition were not reliable for either total or highly confident false recognition of lures from single-item categories (total: *t*_(98)_ = 1.18, *p* = 0.24; confident: *t*_(98)_ = 0.66, *p* = 0.51), but either approached significance or were highly significant for large category abstract lures (total: *t*_(98)_ = 1.77, *p* = 0.08; confident: *t*_(98)_ = 3.52, *p* = 0.001).

## DISCUSSION

The current study investigated whether age-related increases in false recognition are driven by overlapping semantic information between studied items and lures (Koutstaal et al., 2003), or whether as suggested by models of pattern separation decline, OAs are impaired in discrimination along multiple dimensions of similarity (Wilson et al., 2006; Stark et al., 2010). In two experiments, age differences in false recognition of exemplars of previously studied categories of concrete and abstract images were examined, replicating, and extending Koutstaal et al.’s (2003; Experiment 2) earlier study. Despite equivalent or greater veridical recognition of studied items, OAs were less able than YAs to discriminate between studied items and similar lures, showing heightened lure false recognition. The age-related increase in false recognition was particularly evident for concrete images, in line with results of Koutstaal et al. (2003), and as predicted by their semantic categorization account which proposes that OAs emphasize semantic information at encoding, to the detriment of item-specific information. However, in contrast with Koutstaal et al.’s (2003) findings, abstract lure false recognition was also significantly increased in older relative to YAs in both experiments, particularly when multiple abstract category exemplars had been encountered at study. This is consistent with an age-related reduction in mnemonic discrimination across multiple dimensions of similarity, as predicted by models of pattern separation decline (e.g., Yassa et al., 2011a). We consider below how semantic gist and pattern separation views can account for these results.

Findings of larger effects of age on false recognition of concrete images are consistent with proposals that semantic overlap leads to particularly heightened false recognition in OAs (Koutstaal et al., 2003). OAs’ increased false recognition of concrete relative to abstract lures was a robust finding in both experiments and was reflected in highly confident as well as overall recognition responses, again replicating Koutstaal et al.’s (2003) earlier study. In Experiment 2, the requirement to explicitly classify lures as “similar” led to reduced overall false recognition, but this effect was equivalent in both age groups (Koutstaal et al., 1999a), and importantly did not affect the magnitude or direction of the critical Age × Stimulus Type interaction.

We found evidence of increased veridical as well as false recognition in older relative to YAs for large category concrete items, implying comparable effects of semantic similarity on both true and false recognition in older age. In this regard our findings differ from those of Koutstaal et al. (2003), who report non-significant age effects on true recognition, with trends to a slight reduction in veridical recognition in OAs. However, parallel effects of gist on veridical and false recognition are predicted by Fuzzy Trace Theory and other gist accounts, which assume that gist traces can support both veridical and false memory, but that verbatim traces are often required for veridical memory (Koutstaal and Schacter, 1997; Brainerd and Reyna, 2002; Verfaellie et al., 2002). According to gist accounts, including the semantic categorization account, encoding of multiple meaningful exemplars leads to stronger semantic gist representations (Koutstaal et al., 1999a). If OAs rely to a greater extent on gist, this should result in the tendency we observed to endorse more studied items as well as semantically similar lures as “old” on the basis of accurately recognized semantic gist. This is also consistent with evidence that intact semantic knowledge can facilitate episodic memory in older age (Reder et al., 2007; see Umanath and Marsh, 2014 for review). From a pattern separation perspective, presence of multiple overlapping representations in memory (as when multiple similar category exemplars have been encoded), results in increased likelihood of pattern completion of further similar representations, particularly in OAs (Wilson et al., 2006). This too may result in age-related increases in both true and false recognition. Unlike gist accounts however, pattern separation models as currently specified do not predict a specific impact of semantic overlap on true or false recognition.

Despite replication of Koutstaal et al.’s (2003) findings regarding differential effects of age on concrete and abstract false recognition, our findings diverge from theirs in another important respect. In the present study OAs showed reliably increased confident false recognition of abstract lures in both experiments, as well as increased total false recognition of abstract lures in Experiment 2. These effects were smaller than those among concrete items, and were restricted to abstract lures for which multiple category exemplars were presented at study. In analyses collapsing across both experiments, the age effects were highly robust for confident responses. This difference between the two studies may be due to increased power in the current experiments (Experiment 1: *n* = 24 per age group; Experiment 2: *n* = 26, combined analysis: *n* = 50; Koutstaal et al., 2003; *n* = 18). Koutstaal et al. (2003) found numerically (though not significantly) greater large category abstract false recognition in OAs, and referred to unpublished data showing a similar pattern. It is therefore likely that our findings reflect a genuine trend toward heightened false recognition of large category abstract lures, and this suggests age-related increases in false recognition may not be entirely driven by greater reliance on semantic information. This finding presents a challenge to the semantic categorization account, and suggests that OAs may show pervasive effects of similarity on memory, consistent with a decline in pattern separation along multiple dimensions. However, the pattern separation account as currently specified does not explain why age-related differences for abstract items were restricted to large category lures.

One possibility is that OAs indeed rely more on gist representations than YAs but that these gist representations extend beyond the semantic domain. Representations of image perceptual features (e.g., overall shape, color) can form a perceptual gist representation corresponding to the average of an image’s global perceptual features (Oliva, 2005; Oliva and Torralba, 2006). Perceptual similarity between exemplars of abstract categories can drive false recognition in a similar manner to semantic similarity (Koutstaal et al., 1999b; Budson et al., 2001), as can phonological similarity (Budson et al., 2003), suggesting OAs may be more vulnerable to interference from non-semantic as well as semantic gist (Koutstaal and Schacter, 1997). Our false recognition findings are consistent with this: OAs showed larger effects of category size on false recognition than YAs, across both stimulus types. However, alternative possibilities must be considered in interpreting findings for abstract lure false recognition. One is that repeated exposure to abstract category exemplars may prompt formation of a concept for these categories, i.e., they become meaningful. This is consistent with the observation that age differences in abstract false recognition emerged only for large categories. The null result for single abstract items might however, have reflected a floor effect, or a failure on the part of OAs to perceive the similarity between studied items and lures without presentation of multiple exemplars, consistent with their lower ratings of perceptual similarity among abstract items.

A related alternative is that abstract images resembled real-world objects sufficiently to drive increased false recognition directly through increased semantic categorization (Koutstaal et al., 2003). Although abstract categories were designed and normed to be novel and not conceptually meaningful (Koutstaal et al., 2003), our ratings data demonstrated that raters were able to assign conceptual labels to abstract categories to some degree, with modest inter-rater agreement in conceptual labels (0.23). These explanations are compatible with the semantic categorization account, in that semantic gist representations are either formed for, or extracted from, abstract category exemplars, and both may have contributed to current findings.

Regardless of whether non-semantic overlap played a role, enhanced veridical and false recognition for concrete items is assumed to reflect their stronger and pre-existing conceptual representations, resulting in stronger or more readily extracted gist representations (see Umanath and Marsh, 2014). Our replication of Koutstaal et al.’s (2003) finding of larger age differences among concrete items suggests that age-related increases in false recognition are driven principally by strong, pre-existing, rather than recently formed or weaker, semantic representations (Buchler and Reder, 2007). Future studies examining mnemonic discrimination in young and OAs before and after learning of membership criteria for novel categories of objects, e.g., “species” of *greebles* and *fribbles* (Gauthier and Tarr, 1997; Barry et al., 2014), compared with familiar categories, may aid in elucidating whether pre-existing conceptual representations are necessary to elicit age-related deficits in discrimination, and whether formation of a concept leads to similar patterns. It may also be informative to examine age-related differences in the relative influence of semantic and perceptual similarity on false recognition for further classes of stimuli, e.g., scenes, words, and faces (see Smith et al., 1990; Ly et al., 2013; Lee et al., 2014), particularly in light of recent evidence that OAs show a benefit of prior experience in discrimination of perceptually similar faces (Lee et al., 2014).

A caveat to the present findings is that the ratings sample judged concrete items as more perceptually similar than abstract items, unlike raters in Koutstaal et al.’s (2003) iterative procedure used to match complexity and perceptual comparability between abstract and concrete items. Although this discrepancy may be due to differences between ratings samples, it is perhaps more likely that here, despite ratings task instructions, concrete items were rated as more perceptually similar due to their being both perceptually and conceptually similar. In future studies it will be important to reduce the correlation between ratings of the two dimensions, for example using separate ratings of color and shape similarity (Konkle et al., 2010). It is important to note that older raters were not more prone to perceive category members as more similar than YAs; the only age difference in ratings was that YAs rated abstract stimuli as more perceptually similar than OAs. This implies that current findings are not attributable to OAs being less able to discriminate similar items perceptually.

As outlined in the Introduction, pattern separation models predict age-related decline in discrimination ability across multiple dimensions of similarity, but previous investigations of mnemonic discrimination using “old/similar/new” recognition tasks have typically employed meaningful stimuli or familiar shapes. Use of this task with concrete and abstract stimuli in Experiment 2 permitted examination of whether age-related reductions in lure correct rejection and increases in false recognition in this task depend on conceptual overlap. Consistent with age-related decline in discrimination across multiple dimensions, findings of greater overall correct rejection performance among YAs did not reliably differ according to stimulus type, suggesting OAs were impaired in discrimination of both concrete and abstract images. Across both levels of confidence, there were numerically larger effects of age (14 vs. 5.7%) on correct rejection of concrete lures, which may suggest a tendency to a greater age-related reduction in discrimination of meaningful items, although the Stimulus Type × Age interaction was not significant. However, this tendency was driven by effects for the single categories, as reflected in the 3-way interaction for highly confident correct rejection. Unlike for false recognition, OAs were equally impaired in correctly rejecting abstract and concrete lures if multiple category exemplars had been encountered. When a single category exemplar had been studied they were significantly more impaired in concrete than abstract correct rejection. However, this latter finding should be interpreted with caution as lure rejection as “similar” was at floor in both groups in the single category abstract condition. Therefore the data are not conclusive with respect to whether semantic overlap had parallel effects on lure rejection and false recognition, but as in the pattern separation studies, overall age-related differences were present for both.

Parametric measures of perceptual and conceptual similarity permitted testing of a specific prediction of the pattern separation account; that OAs require greater reduction in similarity before lures can be successfully discriminated (Wilson et al., 2006). Findings for concrete items were in line with this: OAs showed greater false recognition than the young for lures with high and medium conceptual (Experiment 1) and conceptual and perceptual (Experiment 2) similarity to studied items, while for the most distinctive lures, false recognition did not differ according to age. Although the predicted pattern dominated for false recognition, group differences were present at all levels of perceptual similarity in Experiment 1. In Experiment 2, group differences for lure correct rejection also did not follow the predicted pattern. Across both age groups, overall effects of perceptual and conceptual similarity on concrete false recognition were of comparable magnitude, but as conceptual and perceptual ratings were correlated among concrete items, it is difficult to determine whether the reduction in the effectiveness of pattern separation was driven by one or both dimensions of similarity. We also note that although planned tests followed practice in the earlier pattern separation studies (e.g., Lacy et al., 2011), effects were relatively modest, an interaction of Similarity with Age being observed only in Experiment 2. Future studies using a similar manipulation can maximize ability to detect age differences in effects across the range of possible item similarity by using a larger number of levels of input similarity (Lacy et al., 2011; Reagh et al., 2014). It would be of interest also to examine mnemonic discrimination of abstract and concrete lures parametrically varied in perceptual features such as angle of rotation or spatial location (Stark et al., 2010; Motley and Kirwan, 2012).

Lack of clear similarity effects for abstract items in both experiments may be due to a combination of substantial variance in abstract false recognition at each level of similarity (compared to concrete lures), very low rates of false recognition of single category abstract lures, and the need to combine single and large category items for similarity analyses to obtain sufficient trials in each bin. However, similarity effects for concrete items are generally consistent with the pattern separation prediction that OAs requires greater reduction in similarity in order to successfully discriminate lures from studied items. It should be noted that similarity ratings were based on raters’ perception of the perceptual/conceptual similarity of all thirteen exemplars presented concurrently, whereas for participants in the recognition experiments, representations of within-category similarity were formed gradually over the course of the study phase. It is possible that greater correspondence between subjectively rated similarity and false recognition rates, and thus clearer age differences, would be obtained if ratings were based on pairs of images and their corresponding lures.

Our findings contrast with those of Ly et al. (2013), who report age-related deficits in mnemonic discrimination of perceptually (phonologically) but not conceptually similar words. However, this apparent discrepancy may be due to use of a different measure of lure discrimination. Ly et al. (2013) examined age differences in the proportion of “new” responses to lures minus the proportion of “new” responses to studied items, a measure which, as dichotomous old/new responses were employed, could not differentiate “new” responses to lures resulting from forgetting of studied items from those resulting from their successful discrimination as similar and therefore “new.” We instead opted to examine novel-corrected false recognition and correct rejection measures which are more typically employed in studies of false recognition (e.g., Schacter et al., 1997; Abe et al., 2011) and pattern separation (e.g., Yassa et al., 2011a; Stark et al., 2013), respectively, and which are arguably more able to isolate the cognitive process under examination (i.e., unsuccessful or successful mnemonic discrimination, respectively). It in fact appears from raw results reported by Ly et al. (2013) that examination of novel-corrected false recognition would reveal a trend in the opposite direction, with OAs showing numerically increased conceptual false recognition, and perceptual false recognition reduced relative to YAs.

The current results are largely consistent with predictions derived from models of declining hippocampal pattern separation ability in older age (Wilson et al., 2006). However, we did not measure neural pattern separation directly: future neuroimaging investigations are needed to assess modulation of hippocampal and cortical functional activity by semantic and non-semantic overlap independently, and assess whether this changes with age. Converging neuroimaging studies are also essential to test predictions about the specific roles of pattern separation and completion during episodic encoding and retrieval (Yassa and Stark, 2011). This would also aid in clarifying whether our findings reflect age-related differences during initial encoding of category exemplars, during the explicit retrieval phase, or both, and in testing specific predictions that semantic categorization at encoding is more pronounced in OAs (Koutstaal et al., 2003). Although behavioral studies are relatively poor at distinguishing between encoding and retrieval effects (Fletcher et al., 1997), task manipulations unique to each phase such as those used by Koutstaal et al. (2003; Experiment 1) may also yield useful information about the locus of age-related differences.

According to dual process models of recognition, OAs are impaired in recollection, and rely to a greater extent on a general feeling of familiarity (Yonelinas, 1999). It has been proposed that strengthened gist representations lead to increased familiarity (Yonelinas, 2002; Duarte et al., 2010), and that increased gist-based false recognition with age reflects greater influence of familiarity (Koutstaal et al., 1999a; Pierce et al., 2005; Dennis et al., 2014). However, medial temporal lobe amnesics showing intact familiarity alongside severely impaired recollection (Turriziani et al., 2008; Addante et al., 2012) have demonstrated reduced conceptual and perceptual gist-based false recognition relative to age-matched controls (Schacter et al., 1997; Verfaellie et al., 2005) suggesting gist-based false recognition is associated with recollection rather than familiarity (Dodson and Krueger, 2006). Consistent with this, conceptually driven false recognition in older age has been more strongly linked to recollection than familiarity (Schacter et al., 1997; Dennis et al., 2014). In YAs, there is increasing evidence of the importance of misrecollection in false memory paradigms (see Arndt, 2012 for review), and a recent study showed lure false recognition in a mnemonic discrimination task to be largely mediated by recollection (Kim and Yassa, 2013). Although an imperfect measure of recollection (Wixted and Mickes, 2010), confident responses are more strongly linked to recollection than familiarity (Mickes et al., 2009). In the current study, as in Koutstaal et al.’s (2003) experiment, age differences in false recognition were substantial and robust for high confidence responses as well as overall, suggesting the impact of semantic interference on age-related increases in false recognition cannot be explained by increased reliance on familiarity, and instead may be mediated by recollection.

Our results support the view that OAs are more susceptible than YAs to memory errors based on conceptual, and to a lesser extent, perceptual gist. They suggest that explicit semantic categorization cannot fully explain this effect, although it may contribute to gist formation. As indicated in the Introduction, although gist accounts assume a central role of semantic similarity in driving increased false recognition with age, while pattern separation models currently do not, the two types of account are likely complementary in other respects (Schacter et al., 1998; Yassa et al., 2011a). A synthesis between these views may explain our findings of substantial age differences in both false recognition and correct rejection of concrete lures, as well as OAs’ greater sensitivity to input interference, and greater false recognition of abstract lures for which multiple perceptually similar images have been viewed. For meaningful items, pre-existing conceptual representations are assumed to be supported by traces stored in semantic memory. Wilson et al. (2006) proposed that prior memories contribute to OAs’ bias toward pattern completion. The present results suggest the presence of semantic overlap between incoming and existing representations may specifically enhance this bias. This would however, not apply in the same way to abstract items, which possess no or few links to existing traces and as such may be less likely to drive pattern completion. However, if recent encoding of multiple similar abstract items permits formation of a perceptual or weak conceptual gist representation, the resulting overlapping traces may drive pattern completion, particularly among OAs. This view is consistent with the complementary learning systems account of memory, which describes the hippocampus as engaged in pattern separation, while the neocortex extracts commonalities between episodes by integrating overlap over experiences (McClelland et al., 1995; Norman and O’Reilly, 2003). As hippocampal function declines with age, the older brain may be more likely to rely on neocortical contributions to memory, emphasizing overlap with previous episodes via pattern completion (Wilson et al., 2006; see also Buchler and Reder, 2007).

## CONCLUSION

In the present study, OAs showed impaired mnemonic discrimination, evidenced by reduced lure correct rejection and heightened lure false recognition. These impairments were particularly heightened when lures were conceptually as well as perceptually similar to studied items. However, increased false recognition was also observed for abstract lures for which multiple perceptually similar images had been viewed. Convergent patterns of results were observed in a typical “old/new” recognition paradigm and an “old/similar/new” recognition task. Our data support the view that OAs are particularly vulnerable to conceptual similarity, as proposed by the semantic categorization account, but are not fully consistent with this view. They also suggest that their false recognition may be increased by perceptual or conceptual gist representations formed for previously unfamiliar “abstract” items. In line with predictions that OAs require greater change in input to successfully pattern separate similar representations, age-related increases in concrete item false recognition were most likely to be observed for highly perceptually or conceptually similar lures, but OAs often performed at the same level as YAs for the most distinctive lures. Together, findings are consistent with a view that the shift in the older brain from a tendency for pattern separation toward pattern completion of input is particularly evident where strong, easily extracted similarities exist between incoming and existing traces, as in the case of frequently encountered common concepts.

## Conflict of Interest Statement

The authors declare that the research was conducted in the absence of any commercial or financial relationships that could be construed as a potential conflict of interest.
